# Comprehensive Characterization of Carbonaceous Material Derived from Rice Husk Pyrolysis and Its Potential for CO_2_ Adsorption

**DOI:** 10.3390/ma18225151

**Published:** 2025-11-13

**Authors:** Santiago Mesa, Javier Ricardo Castro-Ladino, Sandra Liliana Amaya, Cecilia Manrique, Adriana Echavarría, Dora A. Hoyos-Ayala, Laura Uran

**Affiliations:** 1 Grupo de Catalizadores y Adsorbentes (CATALAD), Instituto de Química, Facultad de Ciencias, Exactas y Naturales (FCEN), Universidad de Antioquia UdeA, Cl. 70, No. 52-21, Medellín 050010, Colombia; alba.manrique@udea.edu.co (C.M.); adriana.echavarria@udea.edu.co (A.E.); dora.hoyos@udea.edu.co (D.A.H.-A.); 2 Grupo de Investigación en Tecnologías Emergentes (GITEM), Universidad de los Llanos, Villavicencio 500001, Colombia; jrcastrol@unillanos.edu.co; 3 Grupo de Investigación e Innovación en Energía (GIIEN), Institución Universitaria Pascual Bravo, Facultad de Ingeniería, Cl. 73, No. 73a-226, Medellín 050034, Colombia; sandra.amaya@pascualbravo.edu.co

**Keywords:** carbonaceous material, rice husk, pyrolysis, CO_2_ adsorption, oxygenated function group

## Abstract

In this study, a carbonaceous material was obtained from the thermal decomposition of a non-pretreated rice husk in a pyrolysis system with controlled nitrogen at 700, 800, 900, and 1000 °C. The characterization of the material was performed using various analytical techniques. The results of these characterizations indicate that the obtained carbonaceous material can achieve a surface area of 450 m^2^/g, with a microporous volume of 0.15 cm^3^/g. Furthermore, the presence of oxygenated function groups, predominantly hydroxyl (C-OH) and epoxy (C-O-C), along with amorphous silicon, was identified. Additionally, the material’s CO_2_ adsorption capacity was assessed, revealing a maximum capacity of 1.0 mmol/g. The findings of this study suggest that the CO_2_ adsorption effectiveness can be impacted by the presence of specific functional groups. These groups have been shown to enhance the material’s affinity for CO_2_, along with its porosity and surface area. In this sense, a notable correlation was identified between the oxygenated function group content and CO_2_ adsorption capacity. Also, the adsorption isotherm modeling showed an excellent fit to the Langmuir model, indicating monolayer adsorption on a homogeneous surface.

## 1. Introduction

Rice husk (RH) is an abundant agricultural byproduct generated during the processing of paddy or green rice to produce white rice. It consists of approximately 75% organic matter (primarily lignin, cellulose, and hemicellulose) and 25% ash [1]. Global RH production is estimated at around 150 million tons annually [2], and if not treated properly, this byproduct can generate negative effects on the environment. However, it has interesting compositional characteristics that have attracted significant interest for its potential as a low-cost precursor to produce carbon-based and hybrid materials for diverse applications. These applications include the production of ceramics and concrete [3], chemical adsorption and environmental remediation [4,5,6,7], thermal insulation [8], carbon capture [9], catalysis [10] and energy applications [11,12].

In this context, various approaches for obtaining value-added materials derived from rice husk have been adopted. Generally, two main routes have been proposed [3]: (1) the production of ash from rice husk, primarily involving the extraction of silica through the combustion of biomass, and (2) the pyrolysis of rice husk to obtain carbon-based materials. In general terms, both routes require preliminary treatment processes for the rice husk to remove undesirable components in the final material [13], such as chemical leaching using acidic or basic solutions [14,15,16]. Both valorization routes offer advantages depending on the final application of the material.

In this regard, silica ashes derived from rice husk have found their niche in the cement industry, composites, and reinforcements [17,18]. On the other hand, carbon-based materials have been geared toward applications related to absorbent materials, environmental technologies, and other emerging applications [19,20]. In this context, these carbonaceous materials exhibit a highly porous structure and favorable physicochemical properties, making them particularly well-suited for adsorption processes. Given these considerations, CO_2_ adsorption has emerged as a promising application for carbon-based materials derived from rice husk [21] due to their high porosity, large surface area, and the possibility of functionalizing their surface. These characteristics contribute to their potential effectiveness in CO_2_ capture and storage, with studies demonstrating their promising performance in this area [22,23,24]. For example, chemically activated RH-based carbons have shown CO_2_ adsorption capacities comparable to other adsorbents [24,25], offering a viable alternative with a significantly lower environmental impact.

Despite the promising results, many existing studies involve complex or resource-intensive pretreatment and activation steps [26,27,28,29]. Therefore, there is a continued need to explore simpler and more accessible methods for producing functional RH-derived materials for CO_2_ capture, particularly those that reduce chemical use while preserving adsorption efficiency.

In this work, we developed a straightforward method to produce carbonaceous materials from the pyrolysis of untreated rice husk, avoiding any chemical pretreatment. We performed an extensive characterization of the resulting material to investigate its structural, textural, and chemical properties. Also, focus was placed on correlating these properties (such as surface area, porosity, and surface functionality) with CO_2_ adsorption performance. This approach contributes to understanding the key properties of carbonaceous materials from rice husk pyrolysis and assesses its potential for sustainable carbon adsorbents.

## 2. Materials and Methods

### 2.1. Obtaining Process of the Materials

The raw material, rice husk, was subjected to a sieving process to eliminate remaining dust and dirt. No further treatment was done to the raw material. The rice husk was then placed in a fixed-bed tubular furnace and subjected to thermal decomposition at a heating rate of 10 °C/min until the target temperature was reached. This temperature was maintained for 2 h under a continuous nitrogen flow of 5 L/min to ensure an inert atmosphere. The pyrolysis setup, including the nitrogen-controlled environment, is depicted in Figure S1. By varying the pyrolysis temperature from 700 to 1000 °C every 100 °C, a total of 4 samples were obtained. The carbonization yield information of each pyrolysis temperature is shown in Table S1. The raw husk was characterized by Thermogravimetric analyses (TGA). The materials obtained from pyrolysis were characterized using electron X-ray diffraction (XRD), Fourier Transform Infrared Spectroscopy (FTIR), Raman Spectroscopy, Nitrogen physisorption techniques to establish the surface area and microporosity, Scanning Electron Microscopy (SEM), and X-ray Photoelectron Spectroscopy (XPS).

### 2.2. Characterization

XRD analyses were carried out using a PANalytical Empyrean series II diffractometer (Ni-filtered Cu Kα radiation) (PANalytical, Almelo, The Netherlands). The analysis was performed with a step size of 0.02626 and a scan rate of 0.1117°/s over a 2θ range of 5–85°.

The rice husk was characterized using thermogravimetric analyses (TGA) using a TGA 5500 equipment from TA Instruments (TA Instruments, New Castle, DE, USA). The temperature range was 25 to 1000 °C at a rate of 10°/min under a nitrogen atmosphere.

Scanning-Electron Microscopy (SEM) images were recorded with a JEOL JMS 6490 LV microscope (JEOL, Tokyo, Japan) operated between 15 kV and 20 kV. Transmission electron microscopy (TEM) images were acquired using a Tecnai F20 Super Twin TMP (Thermofisher, Einhoven, The Netherlands). Sample preparation involved dispersing a small amount of the material in ethanol, placing a drop of the suspension onto a copper grid, and allowing it to dry at room temperature. Surface chemistry analysis was carried out by X-ray Photoelectron Spectroscopy (XPS) using a SPECS Surface Nano Analysis GbmH spectrometer (SPECS group, Berlin, Germany) equipped with a monochromate Al-Kα X-ray source (monochromator F600 and excitation energy of 1486.6 eV) and Phoibos hemispherical energy analyzer 150 1D-DLD (SPECS group, Berlin, Germany); spectra were recorded under Ultra-High Vacuum conditions (UHV, at a base pressure of 1 × 10^−9^ mbar) and room temperature (20 °C), operating the spectrometer at 20 W, 13 kV and 0% of spot-size and setting 89.95 eV and 20 eV as energy pass for survey scans and high-resolution (HR) spectra, respectively; all the spectra were calibrated respecting the C1s signal at 284.6 eV.

Raman measurements were carried out at room temperature using a confocal Horiba Jobin Yvon Model Labram HR Raman spectrometer (Horiba, Kyoto, Japan), equipped with an excitation HeNe laser beam operating at a wavelength of 632 nm, with a focal length of 800 mm. All spectra were acquired under the same conditions in a range from 400 to 3500 cm^−1^.

The FTIR measurements were performed using an FT-IR Nicolet iS 50 (Thermo Scientific, Madison, WI, USA). The detection range was 4000–400 cm^−1^ and the optical velocity was 0.474 cm s^−1^. A tablet was prepared to obtain the IR spectra of each sample by preparing a 1:5 mixture of KBr standard.

The nitrogen sorption measurements were performed with a Micromeritics ASAP 2020 gas sorption system (Micromeritics, Norcross, GA, USA) at −196 °C. Prior to the measurements, all samples were degassed under high-vacuum conditions for 6 h at 150 °C. The micropore volume was calculated based on the t-plot method, while the Brunauer–Emmett–Teller (BET) method was applied to determine the specific surface area.

### 2.3. CO_2_ Adsorption Tests

The CO_2_ adsorption test of the materials was evaluated using the TGA method by using a TA Instruments thermogravimetric analyzer (TA Instruments, New Castle, DE, USA). Initially, the sample was subjected to pretreatment at 100 °C, with a heating rate of 10 °C/min, using nitrogen at a flow of 20 mL/min for 60 min. Then, CO_2_ (98%) was introduced in the chamber, and the temperature was decreased to 30 °C, followed by a two-hour isotherm. The amount of CO_2_ adsorbed was determined through the weight variation experienced by the sample during the two-hour isotherm period. In order to establish the thermodynamics parameters, CO_2_ adsorption tests were performed using the same procedure, with a variation in CO_2_ concentration (0, 10, 25, 50, 70 and 100 vol.%, i.e., CO_2_ partial pressures of 0, 10, 25, 50, 70 and 100 kPa) at temperatures of 90 °C, 60 °C and 30 °C. All analyses were performed in triplicate, with a standard deviation of ±0.02.

## 3. Results and Discussion

### 3.1. X-Ray Diffraction and TGA

**Figure 1:**
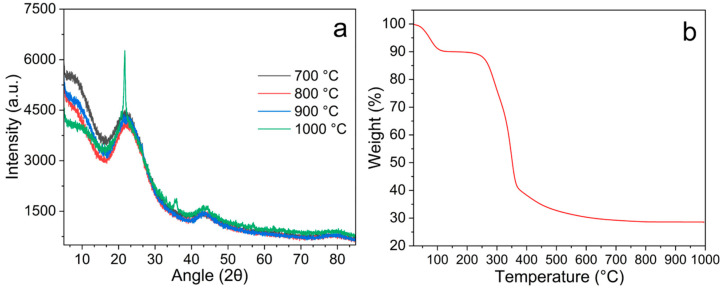
(**a**) XRD patterns of the obtained materials and (**b**) TGA profile of the raw rice husk carried out in N_2_ atmosphere.

The XRD results of the samples are shown in Figure 1a. For the samples obtained at carbonization temperatures of 700, 800, and 900 °C, no peaks indicate the presence of crystalline inorganic compounds. However, the patterns suggest the presence of an amorphous material, identified as amorphous silicon [30]. As the carbonization temperature increased to 1000 °C, XRD analysis showed the appearance of peaks associated with crystalline silica. Typically, the appearance of peaks at two theta values of 21.5°, 35.5°, and 56.9° confirms the formation of crystalline silicon [31]. So, the carbonization process at higher temperatures improved the crystallinity of the silica, resulting in more distinct diffraction peaks.

These results agree with other findings [30,32,33] and are the consequence of the presence of silicon in the rice husk (Table S2). In this regard, the presence of silica in the materials at the temperatures tested in this work is attributed to the high thermal stability of silicon-containing organic compounds, which were not decomposed.

On the other hand, Figure 1b displays the TGA profile of the rice husk under N_2_ atmosphere. A first weight loss in the range 25–100 °C corresponds to the loss of humidity absorbed in the sample. The subsequent weight loss between 120 and 260 °C is correlated to the decomposition of hemicellulose present in the husks. Then, a major mass loss is observed between 260 and 400 °C and is attributed to the breakdown of cellulose. Finally, the lignin is decomposed at temperatures between 350 and 400 °C and is mainly responsible for the char portion of the product [34]. Thermal stability was achieved at 700 °C, confirming that complete carbonization of the material occurred at temperatures above this threshold.

### 3.2. Raman Analysis

**Figure 2:**
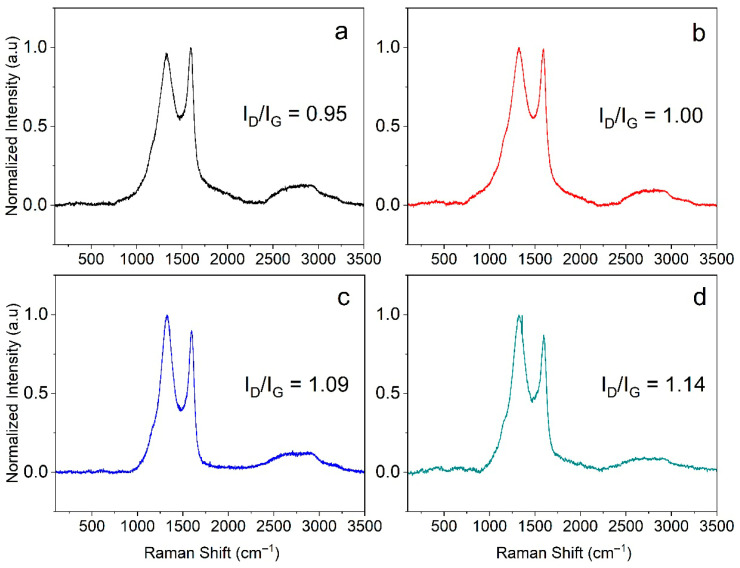
Raman spectra of the samples obtained at (**a**) 700 °C, (**b**) 800 °C, (**c**) 900 °C and (**d**) 1000 °C.

In order to study the carbonaceous portion in the samples, Raman spectroscopy was carried out, and the obtained results are shown in Figure 2. All solids exhibited similar vibrational characteristics. The spectral peaks corresponding to the D, G, and 2D bands appeared approximately at 1328 and 1590, and 2630 cm^−1^, respectively. The D band agreed with the presence of defects; its intensity is proportional to the number of sp^3^ carbon atoms present on the surface of the material and is typically associated with structural defects or disorder [35,36]. The G band is caused by the vibrations of the sp^2^ carbon atoms, representing graphitized carbon and is directly related to the crystallinity of the material. In this regard, the ratio of intensities of the D and G bands (I_D_/I_G_) is used as a measure of disorder of the carbon lattice, i.e., a higher ratio indicates more structural defects [7]. Therefore, the I_D_/I_G_ ratio increased with the activation temperature, revealing that the carbonization temperature favors the formation of structural defects, such as edge sites and imperfections in the carbon network.

### 3.3. FTIR Analysis

**Figure 3:**
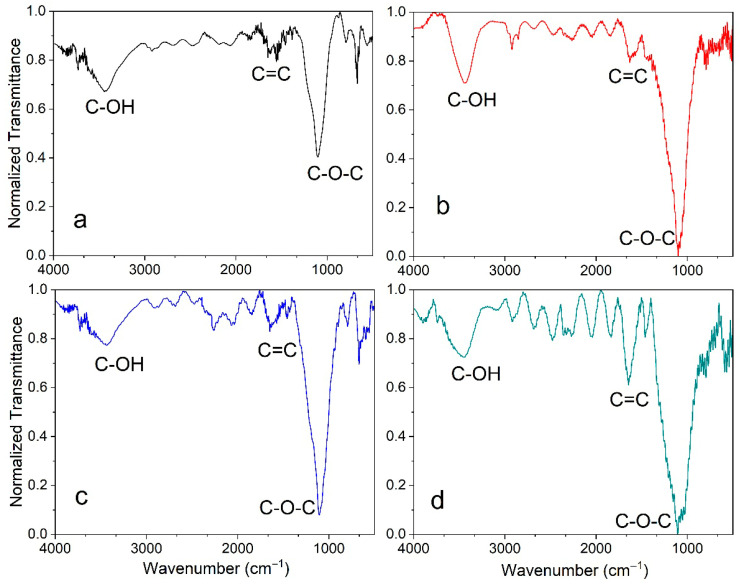
FTIR spectra of the samples obtained at (**a**) 700 °C, (**b**) 800 °C, (**c**) 900 °C and (**d**) 1000 °C.

Another technique employed for the characterization of the functional groups on the material’s surface was Fourier transform infrared (FTIR) spectroscopy. Figure 3 shows the spectra obtained for all the samples. The peak around 3500 cm^−1^ was attributed to stretching vibrations of the hydroxyl group on the surface, while signals around 1600 cm^−1^ and 1200 cm^−1^, corresponding to the stretching vibrations of aromatic C=C and the epoxide group (C-O-C), respectively, were found for all the samples. These findings are consistent with other works [37,38]. Conversely, stretching vibrations of the carbonyl group (C=O) were not observed around 1720 cm^−1^ with this characterization technique, probably due to the low concentration. Additionally, all the spectra showed changes in the signals of C=C and C-O-C with the increase in the temperature, due to the transformation of organic compounds and oxides, resulting in a decrease in C-OH (hydroxyl) bonds and an increase in C-O-C (epoxy) bonds [37].

Based on the above Raman and FTIR results, the observed trend with increasing temperature was the formation of oxygen groups, which could favor CO_2_ adsorption through electrostatic interactions between oxygen groups and CO_2_ molecules [39].

### 3.4. Textural Characterization

**Figure 4:**
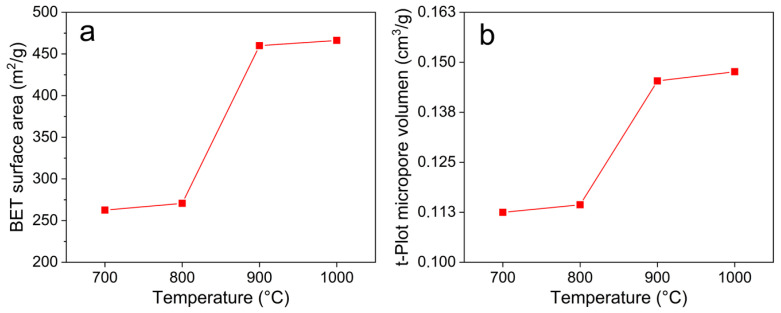
(**a**) BET surface area and (**b**) porosity of the materials obtained at different temperatures.

**Figure 5:**
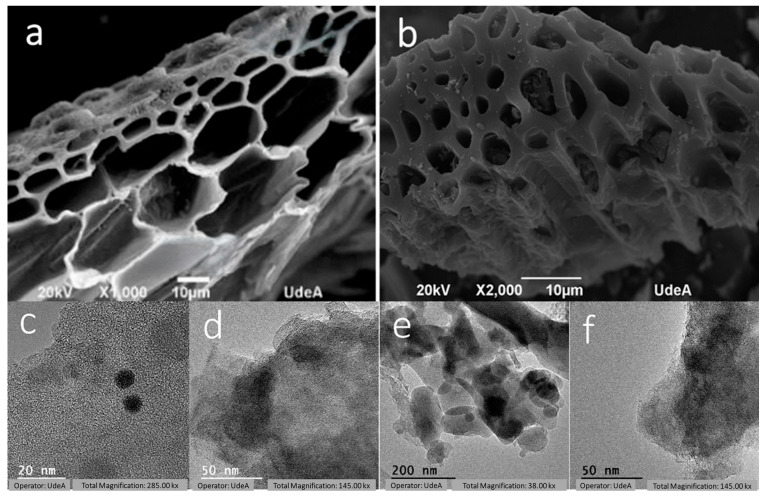
SEM micrographs of materials obtained at (**a**) 700 and (**b**) 1000 °C and TEM images of materials obtained at (**c**) 700, (**d**) 800, (**e**) 900 and (**f**) 1000 °C.

The results of physisorption and subsequent treatment for calculating the BET area are shown in Figure 4a. When the carbonization temperatures were in the range of 700 and 800 °C, the samples exhibited an almost constant average surface area value of 250 m^2^/g. However, when the carbonization temperature rises to 900 and 1000 °C, the surface area increases to around 450 m^2^/g, indicating that the thermal decomposition of lignin and the subsequent stage of formation of the carbonaceous material play a prominent role in the generation of high surface area. Also, the micrographs obtained by scanning electron microscopy (SEM) indicate that with increasing temperature, a thickening of the pore wall was achieved (Figure 5), which also explains the increase in the available surface area. The transmission electron microscopy (TEM) images show that materials obtained at 700 and 800 °C (Figure 5c,d) remain largely amorphous. In contrast, materials treated at 900 °C and 1000 °C (Figure 5e,f) display clustered nanostructures exhibiting signs of crystalline arrangements. These observations suggest that structural ordering improves with temperature, as observed by XRD. Additional TEM images are provided in Figure S2.

On the other hand, the isotherms of the samples exhibit a microporous material behavior, as shown in Figure S3. It was observed that at low pressures, the isotherms exhibit type I behavior due to the microporous characteristics of the material. The initial part of the isotherm at P/P_0_ < 0.2 shows strong Type I adsorption due to the presence of micropores. At medium-to-high pressures (P/P_0_ > 0.2), the behavior transitions to Type IV, featuring a plateau and a hysteresis point characteristic of mesopores [40].

In this regard, the volume of micropores of the materials displayed a similar trend to that of the surface area as a function of the carbonization temperature, as shown in Figure 4b. Also, in Table 1, the textural properties of the materials are depicted. At carbonization temperatures in the range of 700 and 800 °C, the micropore volume was around 0.1 cm^3^/g, without significant variation. However, when the biomass treatment temperature exceeded 900 °C, the micropore volume values increased to around 0.15 cm^3^/g, matching the microporosity volume values reported for hybrid systems obtained with chemical treatments [41,42,43].

### 3.5. XPS Analysis

**Figure 6:**
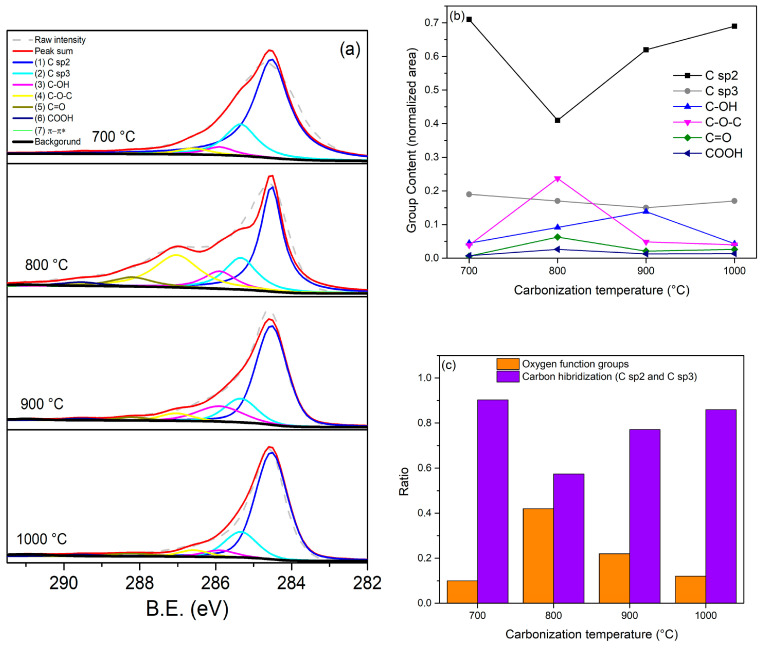
XPS C 1s results: (**a**) XPS C1s spectra; (**b**) oxygen group and carbon hybridization (sp^2^ and sp^3^) trend with carbonization temperature; (**c**) the ratio of oxygen groups (C-OH, C-O-C, C=O, C-OOH) and C atoms of different carbon hybridization (C sp^2^ and C sp^3^).

X-ray photoelectron spectroscopy (XPS) was utilized to assess the presence and variation of the functional groups on the synthesized samples. In this regard, the analysis of the C1s high-resolution spectra elucidated the content of carbon-oxygen groups (hydroxyl, epoxy, carbonyl, carboxyl) and C atoms of different carbon hybridization (C sp^2^ and C sp^3^), as shown in Figure 6 and Table S3. These spectra were fitted by the Gaussian-Lorentzian function, using the XPS peak fit software (XPSPEAK 4.1, freeware program). As illustrated in Figure 6a, the deconvoluted C1s peaks, designated as 1, 2, 3, 4, 5, 6 and 7, correspond to the binding energies of 284.53, 285.34, 285.9, 287.03, 288.2 and 291 eV. These values correspond to C sp^2^ (C=C), C sp^3^ (C-C), hydroxyl (C-OH), epoxy (C-O-C), carbonyl (C=O), carboxyl (COOH), and π–π* satellite bonds, respectively. Additionally, Figure 6b presents the evolution of oxygenated functional groups with carbonization temperature. A general tendency, with a maximum at 800 °C, is observed for all the oxygenated groups, except for the hydroxyl group (C-OH), which continues to increase up to 900 °C and decreases at 1000 °C. This observation indicates the presence of a maximum population of carbon-oxygenated species at 800 °C, where the group epoxy (C-O-C) has the mayor contribution. This hypothesis can be substantiated by Figure 6c, which demonstrates that the ratio of oxygenated species to the total peak area of XPS C1s at 800 °C is maximum with a value of 0.42. In contrast, the C atoms of the different hybridization sp^2^ and sp^3^ have a minimum ratio of 0.57. Conversely, a minimum of oxygenated function groups is observed at 700 °C and 1000 °C, where the normalized peak area of carbon atoms with hybridization sp^2^ reaches its maximum values with 0.71 and 0.69, respectively.

### 3.6. CO_2_ Adsorption Test

**Figure 7:**
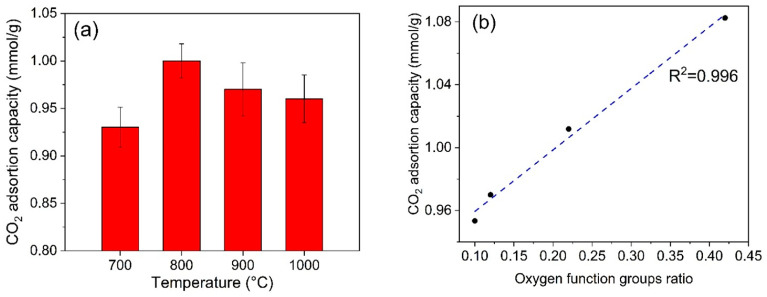
(**a**) CO_2_ adsorption at 30 °C of the obtained materials and (**b**) Relationship between oxygenated function groups content and CO_2_ adsorption capacity.

Figure 7a shows the variation of CO_2_ adsorption capacity (mmol/g) as a function of carbonization temperature of rice husk in the range of 700 °C to 1000 °C, the CO_2_ adsorption data can be seen in Figure S4. The adsorption capacity increases from approximately 0.93 mmol/g at 700 °C to a maximum value of around 1.0 mmol/g at temperatures of 800 and 900 °C. After this plateau, the adsorption decreases to about 0.95 mmol/g at 1000 °C.

The amount of CO_2_ adsorbed would vary in response to changes in the material’s porosity and surface chemical composition as the carbonization temperature increased. The highest adsorptive capacity is observed at 800 °C (1.0 mmol/g), which is consistent with a direct correlation between the volume of micropore (0.114 cm^3^/g) and the presence of functional oxygen groups such as C-OH (0.09) and C-O-C (0.24), which could facilitate the interaction with CO_2_. At temperatures of 900 °C and 1000 °C, there is an increase in the BET area (456.95 m^2^/g and 468.22 m^2^/g) and the total porosity, which could enhance the surface’s accessibility. However, the adsorptive capacity of CO_2_ decreases to 0.97 mmol/g and 0.95 mmol/g, respectively. This may be associated with a change in the pore size distribution, where the formation of mesopores reduces the proportion of micropores that are highly effective for CO_2_ capture. Simultaneously, the functional oxygen groups, particularly C-OH and C-O-C, decrease, indicating a progressive thermal degradation of these active sites. The interaction with CO_2_ would be impacted by the reduction of these groups, which, in conjunction with the increase in area, contributes to the reduced adsorption observed at higher temperatures. These results indicate that the efficacy of CO_2_ adsorption can be influenced by the presence of specific functional groups that can increase the material’s affinity for CO_2_, in addition to porosity or surface area. Therefore, the pyrolysis temperature of 800 °C is the point at which the quantity and type of pores, as well as the presence of functional groups that are suitable for CO_2_ retention, are in equilibrium.

This result is consistent with previous studies that have demonstrated that CO_2_ adsorption in carbon materials primarily occurs in micropores. Mochizuki et al. [40] synthesized activated carbon from a variety of biomasses through pyrolysis and evaluated its CO_2_ adsorption capacity. They found a significant correlation between the specific surface area, micropore volume, and CO_2_ adsorption of the prepared samples, indicating that both the surface area and micropore volume influence CO_2_ adsorption performance.

The presence of oxygen-functional groups on the surface in carbon-based materials has a significant impact on the adsorptive capacity of carbon dioxide. Junkermeier et al. [44] investigated how the adsorption of gases like CO_2_ is affected by the functionalization of carbophenes with carboxyl (COOH), amine (NH_2_), nitro (NO_2_), and hydroxyl (OH) groups. Their results showed that these functional groups can significantly enhance CO_2_ adsorptive capacity in carbon-based materials. Tiwari et al. [45] evaluated oxygen-enriched carbon adsorbents using thermogravimetric analysis at different adsorption temperatures (30–100 °C) and CO_2_ concentrations (6–100%). They found that oxygen-containing groups such as esters, ketones, and ethers can make the surface of carbon more basic and improve interaction with CO_2_ molecules through dipole-quadrupole interactions. Furthermore, they reported that at 30 °C and 100% CO_2_ concentration, the adsorption capacity reached 0.91 mmol/g, demonstrating the positive effect of these functional groups on CO_2_ capture. In our study, we found a direct correlation between the presence of oxygen-functional groups and CO_2_ adsorptive capacity as can be seen in Figure 7b. Specifically, the carbonized material at 800 °C showed a 41% oxygen group content. Therefore, our results suggest that the combination of a high superficial area, high microporous volume, and the presence of oxygenate functional groups could be used as an important parameter for the design of Si/carbon composite active in CO_2_ adsorption.

Moreover, although oxygen-containing functional groups are primarily attributed to the carbonaceous phase, the contribution of oxygenated species derived from silica cannot be entirely ruled out. In composites obtained from rice husk carbonized at high temperatures, SEM/EDS and FTIR analyses have demonstrated the presence of silicon in the form of Si-O-Si and Si-C species intimately integrated within the carbon matrix [46]. This may suggest a direct relationship with the amount of surface oxygenated groups present in the synthetized materials. Therefore, in this study, the presence of both amorphous and crystalline silica phases, as identified by X-ray diffraction, suggests that silanol groups (Si-OH) or Si-O-C linkages may also contribute to CO_2_ adsorption through electrostatic interactions [47].

A review of similar studies on CO_2_ adsorption [20,26,27,48] highlights that the type of biomass precursor, the activation method employed, and the operational conditions—such as temperature and pressure—play a critical role in determining the adsorption performance of these materials. Table 2 presents the CO_2_ adsorption capacities obtained in the present study alongside those reported in the literature. It is worth mentioning that activated carbonaceous materials generally exhibit higher CO_2_ capture capacities compared to non-activated ones. However, the performance of non-activated samples was comparable to that observed in this study. These results confirm the consistency of our findings with those reported in the literature and highlight the promising performance of non-activated carbonaceous materials for CO_2_ capture, particularly in the presence of functional groups that are active in adsorption processes.

**Figure 8:**
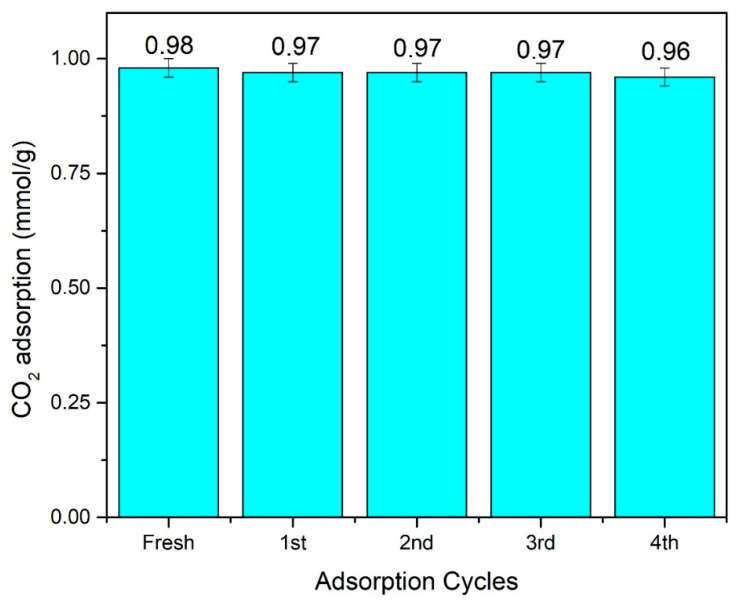
CO_2_ adsorption capacity over consecutive cycles.

The reusability performance of the carbonaceous material was also assessed using the sample obtained at 800 °C. Five consecutive CO_2_ adsorption–desorption cycles were carried out, totaling 500 min, as shown in Figure S5. The CO_2_ adsorption capacities for each cycle, presented in Figure 8, demonstrate that the material maintained a nearly stable uptake throughout the cycles, indicating good regeneration performance and stability of the carbonaceous adsorbent.

### 3.7. Adsorption Isotherms

**Figure 9:**
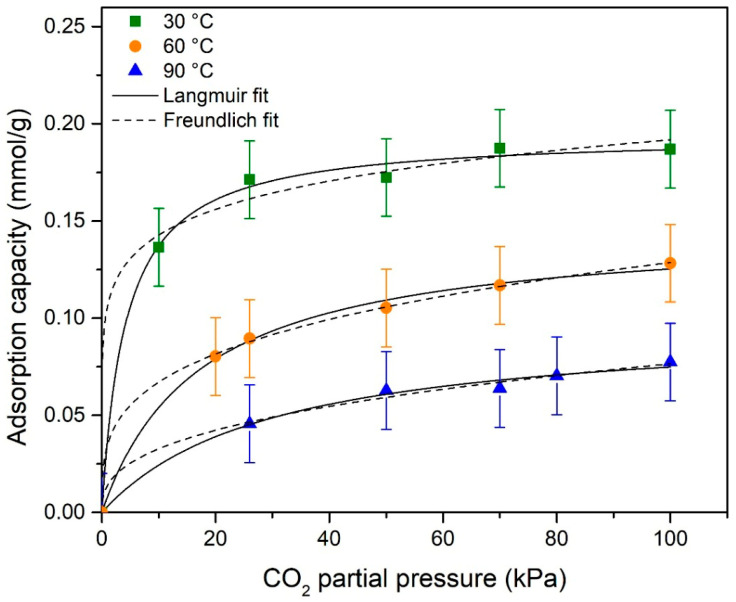
CO_2_ adsorption isotherms with Langmuir and Freundlich models on the carbonaceous material.

The isotherm study was repeated separately at 30 °C, 60 °C, and 90 °C at different CO_2_ partial pressures, for the material obtained at 800 °C. The CO_2_ adsorption data on the adsorbents were fitted to Langmuir and Freundlich isotherm models according to Equations (S1) and (S2). Figure 9 shows experimental data fitting with the Langmuir and Freundlich model for CO_2_ on carbonaceous material. The fit of the curves and the calculated parameters are given in Table 3. When the isotherm parameters calculated were examined, it was seen that the best correlation for CO_2_ adsorption on carbonaceous material from rice husk is better suited to the Langmuir isotherm, suggesting a monolayer adsorption and a homogeneous surface of the adsorbent [50,51].

### 3.8. Adsorption Thermodynamics

The calculated thermodynamic parameters from Equations (S3) and (S4) for CO_2_ adsorption on carbonaceous material are presented in Table 4. The linear fit is shown in Figure S6.

The values of the carbon dioxide adsorption enthalpy change have been determined to be −30.58 kJ/mol. This finding indicates that the CO_2_ adsorption process on carbonaceous material is exothermic, and it is further concluded that this adsorption is physical. The values of Gibbs free energy are positive, which indicates the non-spontaneity of the process at the evaluated temperatures. The decrease in values with decreasing temperature indicates enhanced adsorption at lower temperatures, consistent with the observed increase in adsorption capacity. The negative value of ∆S^0^ suggests that the adsorption process is enthalpy driven [52,53].

## 4. Conclusions

This study successfully demonstrated the production of carbonaceous materials from the pyrolysis of raw rice husk and their application as CO_2_ adsorbents. The characterization results confirmed that the synthesized materials exhibit a combination of graphitized carbon structures with oxygenated functional groups, including hydroxyl (C-OH) and epoxy (C-O-C), which play a crucial role in adsorption performance. Also, the characterization indicated the presence of amorphous silicon. The thermal decomposition process led to significant changes in the physicochemical properties of the materials, with increasing carbonization temperature enhancing surface area and porosity. The highest BET surface area of 468 m^2^/g and a micropore volume of approximately 0.15 cm^3^/g were achieved at 1000 °C. However, these structural parameters alone did not determine CO_2_ adsorption capacity, reinforcing the importance of surface chemistry in adsorption processes.

The CO_2_ adsorption tests revealed that the material synthesized at 800 °C exhibited the highest adsorption capacity of 1.0 mmol/g, and that this CO_2_ adsorption capacity remains stable after five consecutive adsorption–desorption cycles. This performance was attributed to the optimal balance between microporosity and the presence of oxygenated functional groups that facilitate electrostatic interactions with CO_2_ molecules. Although higher temperatures (900 °C and 1000 °C) resulted in increased surface area, the adsorption capacity declined slightly due to the formation of mesopores and a reduction in oxygen functional groups, which reduced CO_2_ binding sites. These results indicate that while textural properties, such as surface area and micropore volume, are essential, the presence of functional groups significantly influences adsorption performance. Therefore, the interplay between porosity and surface chemistry must be carefully optimized to develop efficient carbon-based CO_2_ adsorbents. The modeling of adsorption isotherms revealed that the experimental data exhibited an excellent fit to the Langmuir isotherm model, suggesting a monolayer adsorption mechanism on a homogeneous adsorbent surface. Furthermore, the analysis of thermodynamic parameters indicates that the adsorption of CO_2_ onto the carbonaceous material derived from rice husk is an exothermic and physical process, which is favored at lower temperatures.

Beyond CO_2_ capture, these findings highlight the broader potential of raw rice husk-derived carbonaceous materials for industrial applications. The ability to tailor the material’s surface chemistry and porosity through controlled pyrolysis conditions enables their use.

Overall, this study contributes valuable insights into the synthesis and optimization of biomass-derived carbon materials for a particular application. Future research should focus on further tuning synthesis parameters, exploring chemical modifications to enhance adsorption performance, and assessing the long-term stability of these materials under real-world conditions. By advancing the understanding of structure-property relationships in carbonaceous materials, this work paves the way for more efficient and sustainable approaches to mitigating carbon emissions.

## Figures and Tables

**Table 1:** Textural properties of materials obtained from the N_2_ sorption isotherms.

Sample (°C)	S_BET_^a^	S_micro_^b^	S_ext_^c^	V_total_^d^	V_micro_^e^
700	263	222	40.64	0.112	0.112
800	271	226	44.86	0.114	0.114
900	457	377	80.07	0.220	0.144
1000	468	293	72.03	0.227	0.149

^a^ BET specific surface area (m^2^/g). ^b^ t-plot micropore area (m^2^/g). ^c^ t-plot external surface area (m^2^/g). ^d^ Total pore volume (cm^3^/g). ^e^ t-plot micropore volume (cm^3^/g).

**Table 2:** Comparative table with different biomasses in CO_2_ adsorption.

Sample	Pretreatment	T, P *	Surface Area (m^2^/g)	CO_2_ Adsorption (mmol/g)	Reference
Carbonaceous material from rice husk (800 °C)	Without activation	30 °C, 1 bar	271	1.0	Present work
Corn stover-derived porous carbon (AC800-2-2)	KOH activation	0 °C, 1 bar	2442	7.14	[48]
Corn stover-hydrothermal carbonization (HC)	Without activation	0 °C, 1 bar	11	0.71	
Albizia procera leaves-derived nitrogen-doped carbons (NDCs)	NaHCO_3_ activation	0 °C, 1 bar	426	2.54	[26]
Water chestnut shell	KBO2 activated	0 °C, 1 bar 25 °C, 1 bar	683	4.22 3.15	[49]
Rice husk-derived porous activated carbon	KOH activation	0 °C, 1 bar	755.73	3.13	[20]
		25 °C, 1 bar		2.24	
		50 °C, 1 bar		1.55	
	W/O activation	0 °C, 1 bar	9.77	1.39	
		25 °C, 1 bar		1.06	
		50 °C, 1 bar		0.74	
Rice husk biochar	KOH activation	25 °C, 1 bar	2492.9	3.10	[27]

* T (Temperature), P (Pressure).

**Table 3:** Isotherm parameter values.

Model	Parameter	Temperature (°C)		
		30	60	90
Langmuir	q_m_ (mmol/g)	0.194	0.147	0.097
	K_L_ (kPa^−1^)	0.240	0.058	0.033
	R^2^	0.996	0.997	0.990
Freundlich	K_F_	0.106	0.035	0.014
	*n*	7.805	3.539	2.717
	R^2^	0.854	0.995	0.942

**Table 4:** Thermodynamic parameters ΔH^0^, ΔS^0^ and ΔG^0^ for CO_2_ adsorption.

Temperature (°C)	∆G^0^ (kJ/mol)	∆H^0^ (kJ/mol)	∆S^0^ (kJ/mol K)	R^2^
30 (303.15 K)	3.85	−30.58	−0.11	0.964
60 (333.15 K)	7.26			
90 (363.15 K)	10.67			

## Data Availability

The original contributions presented in this study are included in the article/Supplementary Material. Further inquiries can be directed to the corresponding authors, S.M. and L.U.

## Supplementary Materials

The following supporting information can be downloaded at: https://www.mdpi.com/article/10.3390/ma18225151/s1, Figure S1: Schematic for the process to obtain the composite materials; Figure S2: TEM images of the materials obtained at 700 °C, 800 °C, 900 °C and 1000 °C; Figure S3: N_2_-adsorption/desorption isotherms of the samples obtained at (a) 700 °C, (b) 800 °C, (c) 900 °C and (d) 1000 °C; Figure S4: CO_2_ adsorption capacity in mg; Figure S5: Consecutive CO_2_ adsorption–desorption cycles; Figure S6: Plot ln (K) vs. 1/T; Table S1: Carbonization yields from each pyrolysis temperature; Table S2: XRF analysis of the material obtained at 1000 °C; Table S3: XPS compositional fitted parameters of C1s spectra of prepared samples at different carbonization temperatures.
