# Supplementary material for: Comprehensive Characterization of Carbonaceous Material Derived from Rice Husk Pyrolysis and Its Potential for CO2 Adsorption
Source: Materials (Basel). 2025 Nov 13;18(22):5151. doi: 10.3390/ma18225151 (PMC12654578; doi:10.3390/ma18225151)
Supplement: Supplementary file 1 [file materials-18-05151-s001.zip › materials-3866222-supplementary.pdf]

SUPPLEMENTARY MATERIAL

Comprehensive characterization of carbonaceous material derived from rice husk pyrolysis and its potential for CO_2 adsorption

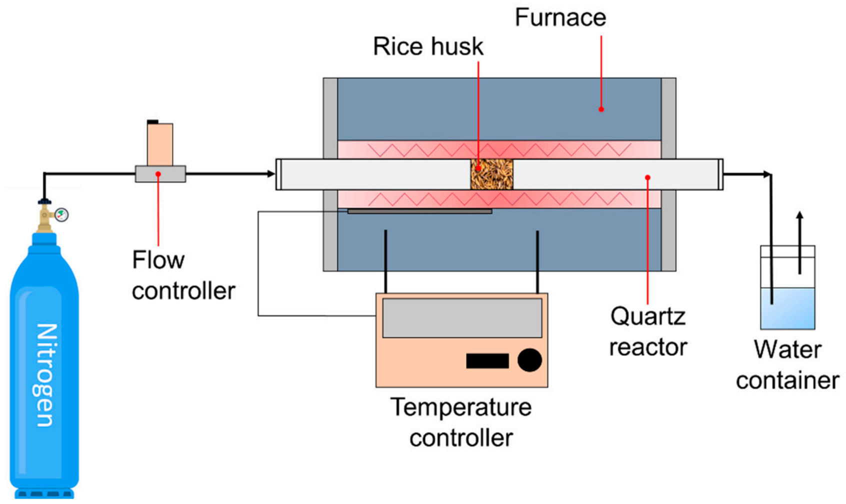

Figure S1 Schematic for the process to obtain the composite materials

Table S1. Carbonization yields from each pyrolysis temperature.

Carbonization temperature (°C)	Raw material received (RH) (g)	Carbon obtained (g)	Carbonization yield (%)
700	20.0067	6.5128	32.55
800	20.0205	6.3701	31.82
900	20.0088	5.2543	26.26
1000	20.0249	5.2646	26.29

Table S2. XRF analysis of the material obtained at 1000°C

Element	Compound	Composition (%w/w)
Silicon	SiO_2	93.29
Aluminum	Al_2O_3	0.53
Iron	Fe_2O_3	1.66
Manganese	Mn_3O_4	0.78
Magnesium	MgO	0.68

Calcium	CaO	0.95
Potassium	K_2O	0.74
Phosphorous	P_2O_5	0.84
Sulphur	SO_3	0.34
Zinc	ZnO	0.19

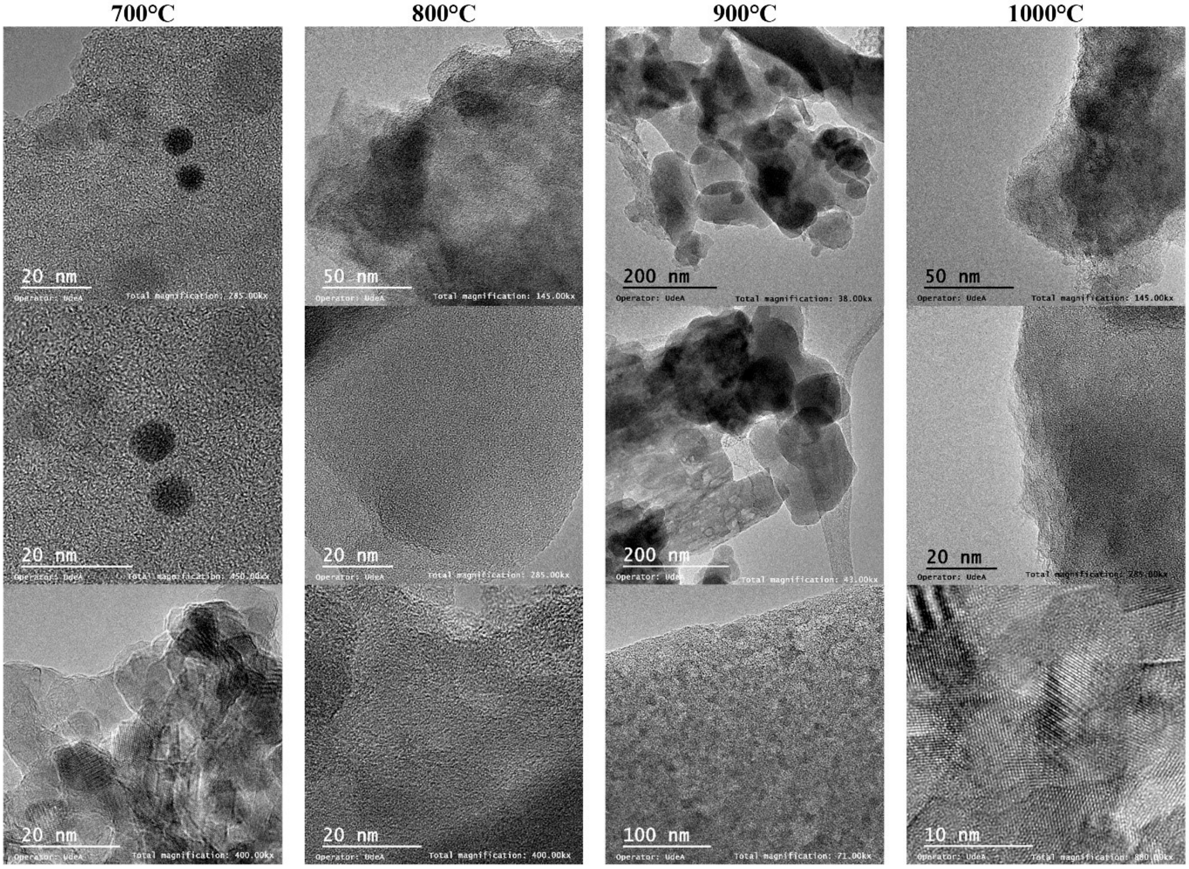

Figure S2 TEM images of the materials obtained at 700°C, 800°C, 900°C and 1000°C

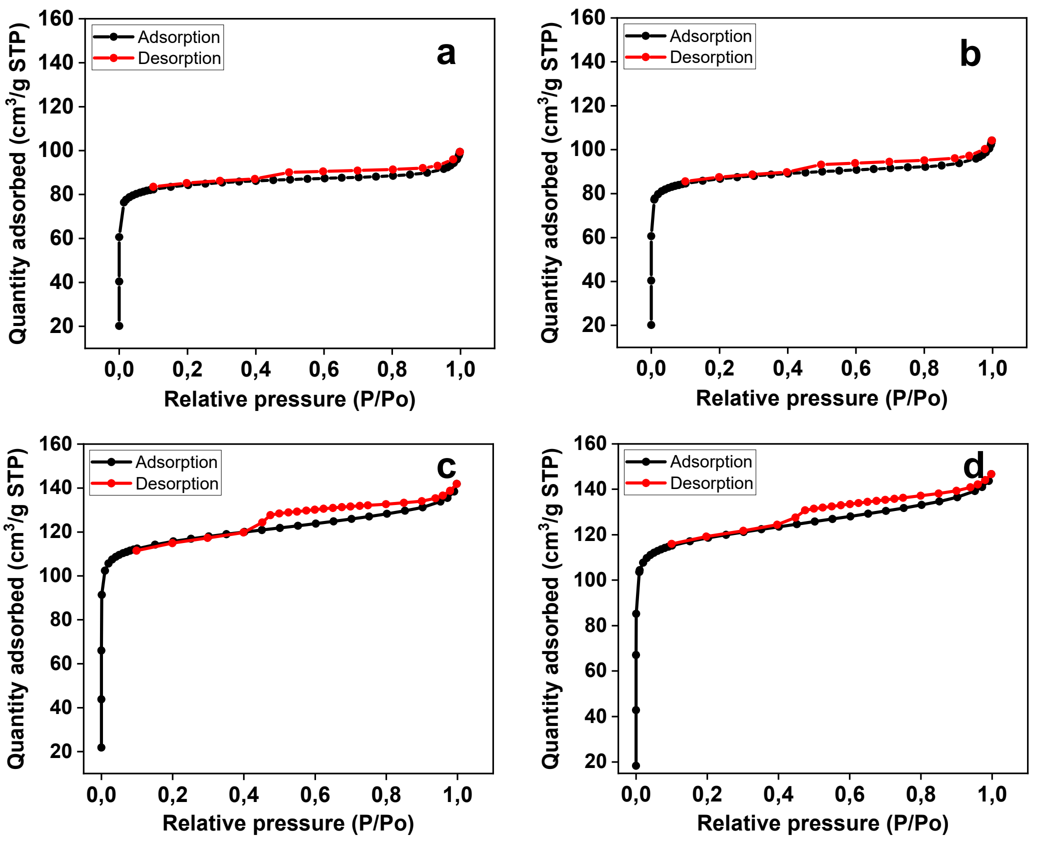

Figure S3 N_2-adsorption/desorption isotherms of the samples obtained at (a) 700°C, (b) 800°C, (c) 900°C and (d) 1000°C

Table S3. XPS compositional fitted parameters of C1s spectra of prepared samples at different carbonization temperatures.

Peak	Phase group	Binding energy (eV)	Normalized Peak area				FWHW (eV)			
			700	800	900	1000	700	800	900	1000
(1)	C sp2	284.53	0.71	0.41	0.62	0.69	1.1	0.7	0.97	1
(2)	C sp3	285.34	0.19	0.17	0.15	0.17	1	1	1	1
(3)	C-OH	285.9	0.04	0.09	0.14	0.04	0.9	1	1.4	1
(4)	C-O-C	286.58	0.04	0.24	0.05	0.04	1	1.4	1	1
(5)	C=O	288.2	0.01	0.06	0.02	0.03	1	1.3	1	1.51
(6)	COOH	289.55	0.01	0.03	0.01	0.01	1	1.3	1	1.18
(7)	π-π^*	291	0	0.01	0.01	0.02	1	1.4	1	1.51

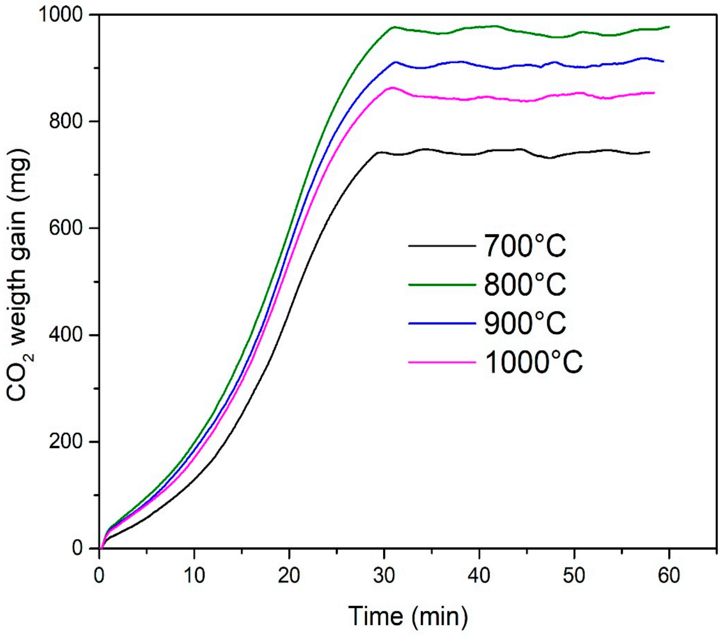

Figure S4. CO_2 adsorption capacity in mg

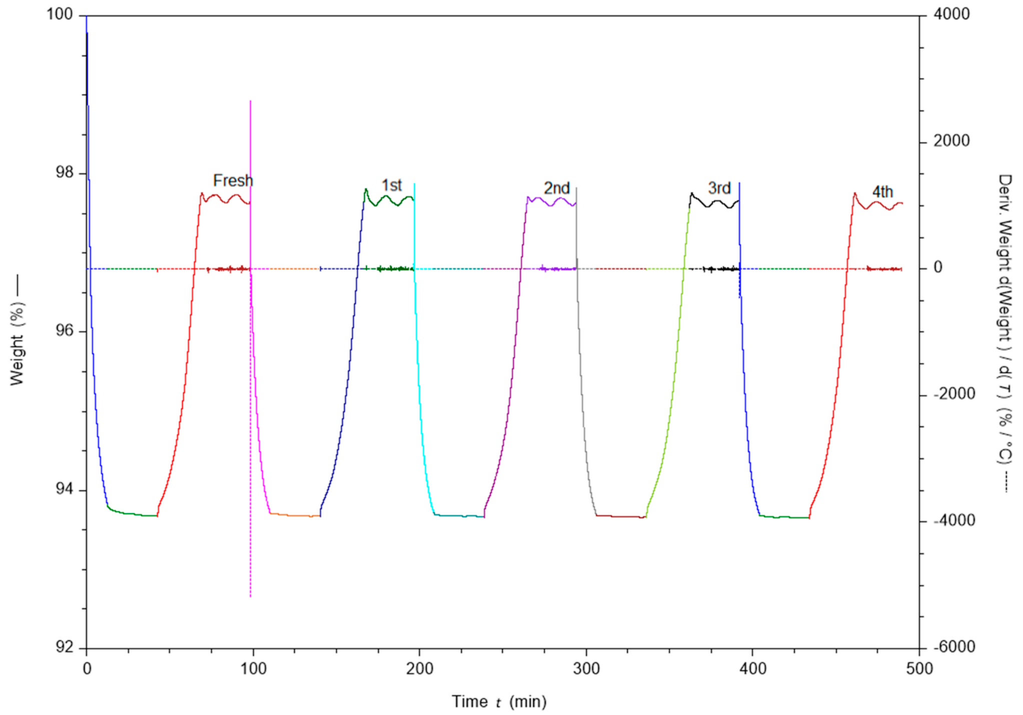

Figure S5 Consecutive CO_2 adsorption–desorption cycles

Adsorption isotherm models

CO_2 adsorption data on the adsorbents were fitted to two standard isotherm models:

The Langmuir equation can be written as [1]:

$$q_e = \frac{q_m K_L P}{1 + K_L P} \tag{S1}$$

Where, q_e is the CO_2 adsorbed (mmol g^{-1}) on carbonaceous material, P is the CO_2 partial pressure in kPa, q_m is the maximum monolayer adsorption capacity (mmol g^{-1}); K_L is the Langmuir constant (kPa^{-1}).

The Freundlich equation can be written as [1]:

$$q_e = K_F P^{1/n} \tag{S2}$$

Where n and K_F are the Freundlich constants revealing the adsorption intensity and capacity respectively. Here, n is the empirical constant associated with the adsorption driving force.

Thermodynamic studies

The standard Gibbs free energy change (ΔG^0, kJ mol^{-1}) was estimated using Equation S3. The standard molar adsorption enthalpy (ΔH^0, kJ mol^{-1}) and the standard entropy change (ΔS^0, kJ mol^{-1} K^{-1}) were determined from the slope and intercept, respectively, of the linear fit shown in Fig. S5, using Equation S4 [1]

$$\Delta G^0 = \Delta H^0 - T\Delta S^0 \tag{S3}$$

$$\ln(K_{eq}) = -\frac{\Delta H^{\circ}}{R}\frac{1}{T} + \frac{\Delta S^{\circ}}{R} \tag{S4}$$

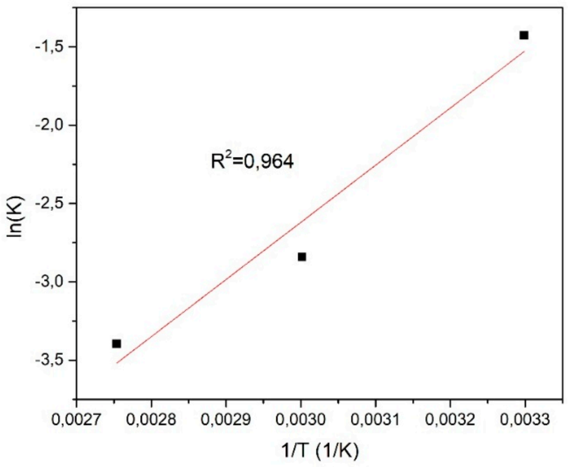

Fig. S6 Plot ln (K) vs 1/T

References

[1] J. Singh, S. Basu, and H. Bhunia, "Dynamic CO2 adsorption on activated carbon adsorbents synthesized from polyacrylonitrile (PAN): Kinetic and isotherm studies," *Microporous and Mesoporous Materials*, vol. 280, pp. 357–366, May 2019, doi: 10.1016/j.micromeso.2019.02.031.
